# Deep learning based DNA:RNA triplex forming potential prediction

**DOI:** 10.1186/s12859-020-03864-0

**Published:** 2020-11-12

**Authors:** Yu Zhang, Yahui Long, Chee Keong Kwoh

**Affiliations:** 1 School of Computer Science and Engineering, Nanyang Technological University, Singapore, 639798 Singapore; 2 College of Computer Science and Electronic Engineering, Hunan University, Changsha, 410000 China

**Keywords:** Long noncoding RNAs, DNA:RNA triplex, Deep learning

## Abstract

**Background:**

Long non-coding RNAs (lncRNAs) can exert functions via forming triplex with DNA. The current methods in predicting the triplex formation mainly rely on mathematic statistic according to the base paring rules. However, these methods have two main limitations: (1) they identify a large number of triplex-forming lncRNAs, but the limited number of experimentally verified triplex-forming lncRNA indicates that maybe not all of them can form triplex in practice, and (2) their predictions only consider the theoretical relationship while lacking the features from the experimentally verified data.

**Results:**

In this work, we develop an integrated program named TriplexFPP (Triplex Forming Potential Prediction), which is the first machine learning model in DNA:RNA triplex prediction. TriplexFPP predicts the most likely triplex-forming lncRNAs and DNA sites based on the experimentally verified data, where the high-level features are learned by the convolutional neural networks. In the fivefold cross validation, the average values of Area Under the ROC curves and PRC curves for removed redundancy triplex-forming lncRNA dataset with threshold 0.8 are 0.9649 and 0.9996, and these two values for triplex DNA sites prediction are 0.8705 and 0.9671, respectively. Besides, we also briefly summarize the *cis* and *trans* targeting of triplexes lncRNAs.

**Conclusions:**

The TriplexFPP is able to predict the most likely triplex-forming lncRNAs from all the lncRNAs with computationally defined triplex forming capacities and the potential of a DNA site to become a triplex. It may provide insights to the exploration of lncRNA functions.

## Background

The advances in sequencing technologies enable the discovery of the vast amount of Long non-coding RNAs (lncRNAs). lncRNAs can serve as signals, decoys, guides, and scaffolds to carry out functions like chromatin states modulation and gene expression regulation. They act via the interactions with DNA, protein, and other RNA, in the way of coordinating regulatory proteins, localizing to target loci, shaping three-dimensional (3D) nuclear organization [1–3], etc*.*

One way for lncRNA to interact with DNA is to form triplex structures [4]. Triplex is a kind of direct RNA–DNA interaction mechanism, it is formed through the binding of RNA sites and purine rich strand of duplex DNA under the forward or reverse Hoogsteen base-pairing rule. Some lncRNAs are proved to execute functions via forming DNA:RNA triplexes, for example, promoter associated lncRNA interacts with TTF-I to repress transcription of rRNA [5], FENDRR increases PRC2 occupancy at the triplex formation sites [6], MEG3 forms DNA-lncRNA triplex with TGF-β gene to modulate the gene activity [7], PARTICLE binds to MAT2A promoter CpG island as triplex to contribute to gene-silencing machineries [8], KHPS1 interacts with SPHK1 to anchor the lncRNA and associated effector proteins to the gene promoter [9], HOTAIR forms triplex with PCDH7 and HOXB2 to regulate adipogenic differentiation [10], MIR100HG acts via triplex formation to regulate p27 [11], and promoter and pre-rRNA antisense guides associated CHD4/NuRD to the rDNA promoter [12].

Although the recent development of high throughput techniques, such as Chromatin Isolation by RNA Purification (ChIRP-seq) [13], capture hybridization analysis of RNA targets (CHART-seq) [14], RNA Antisense Purification (RAP-seq) [15], and chromatin oligo affinity precipitation (ChOP-seq) [7], has helped to generate the genome-wide map of lncRNA chromatin interactions for specific lncRNAs via deciphering their binding sites, most of them are implemented in crosslinked chromatin which contain RNA associated to DNA binding proteins. Therefore, they cannot provide reliable references for the studying of DNA:RNA triplex formation. To reveal the existence of the DNA:RNA triplex interactions in vivo, Cetin et al*.* [16] developed a method to map the genome-wide DNA:RNA triplexes by excluding the chromatin crosslinking . This method proved the physiological relevance of DNA:RNA triplex structures.

Currently, the prediction of DNA:RNA triplex mainly relies on the base paring rules-related mathematic statistics. Triplexator is proposed to systematically identify the potential triplex forming sites of RNA and the targeting sites on DNA by taking the Hoogsteen and reverse-Hoogsteen base-pairing into account [17], Triplex-Inspector is designed to select sequence-specific ligands and targets by considering the gene location and genomic architecture [18], LongTarget is presented to detect motifs and binding sites in forming triplex by considering non-canonical rules [19], and Triplex Domain Finder (TDF) is developed to predict triplexes and characterize lncRNA and the corresponding DNA targets [20].

Although the above methods can identify potential triplexes according to the canonical rules, they predict a large population of lncRNAs with triplex forming potential. However, the limited number of experimentally identified triplex-forming lncRNAs indicates that maybe not all of them can form triplex in practice. Besides, the computational methods only calculate the theoretical triplex potential, while do not consider any in vivo or in vitro assays verified data.

In this work, we have the following two aims: (1) predicting the most likely triplex-forming lncRNAs in practice from the lncRNAs owning triplex forming capabilities calculated by the computational methods, and (2) predicting the potential of DNA sites in forming triplex based on the experimentally verified data. For these purposes, we develop TriplexFPP (Triplex Forming Potential Prediction). It is the first machine learning program in DNA:RNA triplex forming potential prediction according to our knowledge. In triplex lncRNA prediction, the average values of Area Under the ROC (AUROC) and Area Under the PRC (AUPRC) for fivefold cross validation on removed redundancy dataset with threshold 0.8 are 0.9649 and 0.9996 separately. Besides, the average cross-validation AUROC and AUPRC values for the triplex DNA sites potential prediction are 0.8705 and 0.9671 separately. The general good performances of TriplexFPP illustrate its effectiveness in triplex forming potential prediction, and could provide references to the lncRNA function exploration. Furthermore, we also briefly summarize the *cis* and *trans* targeting of triplexes lncRNAs, which may provide some insights to the exploration of lncRNA binding mechanisms.

## Implementation

### Dataset

#### Triplex lncRNA prediction dataset

The positive data for triplex lncRNA prediction is collected in 2 ways. On one hand, we extracted the lncRNAs according to the TriplexRNA regions (DNA:RNA triplex forming peaks in RNA) reported in the work of Sentürk et al*.* [16] by considering both Solid Phase Reversible Immobilization-based paramagnetic bead size selection and immunopurification with anti-DNA antibody RNA separation in Hela S3 cell. We used GENCODE release 33 lncRNA annotation [21] to extract the lncRNAs that cover the TriplexRNA regions and obtained 476 unique samples in this way. We named these lncRNAs as triplexlncRNA. On the other hand, we also collected lncRNAs that are verified by either in vivo or in vitro assays to from triplexes with DNA from the peer-viewed publications. These lncRNAs are named as reported triplex lncRNA, including MEG3 [7], PARTICL [8], MIR100HG [11], FENDRR [22], and HOTAIR [10]. All the variants of the reported triplex lncRNA were taken into consideration. The total number of reported triplex lncRNA is 159.

Since our goal is to predict the most likely triplex-forming lncRNAs in practice from the lncRNAs owning triplex forming capacities predicted by computational methods, we used TDF [20] to further filter the data. When evaluating the triplex forming potential of the above 635 lncRNAs with the whole gene promoters (except for chromosome Y and M) with TDF (default parameters), 104 of them do not contain DNA Binding Domains (DBDs) with powerful Triplex Forming Oligonucleotide (TFO) support. There are two possible explanations for the phenomenon of low triplex forming capacity obtained from TDF in collected positive data: i) for reported triplex lncRNA, each lncRNA gene may have multiple transcripts with splice variants, but maybe not all of their variants have the abilities to form the triplex with DNA, and ii) for triplexlncRNA, the overlap regions between R-Loop and TriplexRNA regions cannot be confirmed as triplex forming or not [16]. To assure the data reliability, we deleted the 104 lncRNAs with low triplex forming capacity and finally got 531 samples in the positive dataset.

The negative samples are collected following the same filter rules as that of positive. After removing the lncRNAs in our original positive dataset from GENCODE annotation, we evaluated the triplex forming potential of all remaining lncRNAs with the whole gene promoters (except for chrY and chrM) by TDF. We only kept the lncRNAs with at least one powerful TFO supported DBD and at least 123 DNA Binding Sites (DBSs) (the smallest number of DBSs is 123 in positive data). From the qualified lncRNAs, we further removed one lncRNA with letter ‘N’ in its sequence and the variants of lncRNA MALAT1 which is reported to form RNA-RNA triplex [23]. Finally, the negative dataset contains 36,021 lncRNAs which have comparable triplex forming abilities as the positive data.

We also prepared two more datasets with removed redundancies. We used the CD-HIT [24] to remove the redundancy in each class of the original dataset with threshold of 0.9 and 0.8 separately. The positive and negative data amounts in two removed redundancy datasets are 384 and 28,012, and 286 and 22,681, respectively.

#### Triplex DNA sites potential prediction dataset

To predict triplex-forming sites in DNA on the basis of experimental data, we adopted the TriplexDNA regions (DNA:RNA triplex forming peaks in DNA) obtained from [16] as positive data. These RNA-associated DNAs are enriched by an unbiased approach. After removing 5 samples containing the letter 'N' in their sequences, the final positive dataset size is 2542. The negative data is selected as the random regions in promoters. We downloaded all ensembl annotated promoters [25], from which we generated 12,735 regions (5 times amount of originally positive data). These regions were obtained by randomly selecting chromosomes (except chrY and chrM) and DNA regions with the same lengths as TriplexDNA. The sequence data for both positive and negative is extracted from the DNA minus strand.

### Feature extraction

Two types of sequence-related feature extraction strategies are considered here: k-mer and kmerscore. Both two types of strategies have been successfully applied to classification problems in RNA [26, 27]. K-mer is a popular method to transform a sequence into a vector, it counts the frequencies of single or multiple nucleotide compositions in a sequence and represents the sequence into a 4^ k^ dimensional vector. K-mer features can be calculated as

1 $$kmer\left( i \right) = \frac{{Total\, number\,of\,k\,neighboring\, nucleic\,acids\,\left( { i} \right)}}{n - k + 1}$$

where $kmer\left( i \right)$ is the frequency of the $i$th nucleotide composition in all 4^ k^ possibilities, and the denominator $n - k + 1$ represents the total number of all possible $k$ neighboring nucleic acids in a sequence with length $n$. For example, in the 3-mer circumstance, $k = 3$, considering sequence $S = AAAAC$, whose $n = 5$, its frequencies under the nucleotide composition of $AAA$ and $AAC$ are $2/3$ and $1/3$ separately, while the frequencies for other 3-mer compositions like $AAG$, et al*.* are $0$.

kmerscore is an overall measure of the k-mer nucleotide composition bias in a sequence, it is obtained from k-mer features. To calculate it, firstly, the k-mer features for all sequences need to be calculated, then the mean k-mer vectors from the positive and negative dataset are obtained from the corresponding k-mer features separately, which are represented as $M_{pos} \left( {h_{i} } \right)$ and $M_{neg} \left( {h_{i} } \right)$, where $i = 1,2, \ldots ,4^{k}$. Finally, for a nucleotide sequence $S = s_{1} s_{2} \ldots s_{n}$ with k-mer sequence $S = h_{1} h_{2} \ldots h_{n - k + 1}$, the kmerscore can be represented as

2 $$kmerscore = \frac{1}{n - k + 1}\mathop \sum \limits_{i = 1}^{n - k + 1} log\frac{{M_{neg} \left( {h_{i} } \right)}}{{M_{pos} \left( {h_{i} } \right)}}$$

### Model construction

We developed an integrated machine learning program called TriplexFPP (Triplex Forming Potential Prediction) in triplex forming potential prediction. It consists of two individual models, including triplex lncRNA prediction model and triplex DNA sites potential prediction model.

We adopted the 2-layer Convolutional Neural Network to construct the models, which can effectively learn the high-level features. The detailed description of the structure and parameters of the model can be found at section of Initial training of TriplexFPP below. As the positive dataset sizes and negative dataset sizes in triplex lncRNA prediction and triplex DNA sites prediction are imbalanced with ratios around 1:68 and 1:5 separately, to avoid the model bias, we applied the random down-sampling technique during the training process. The negative training data were randomly selected as the same amount as positive training data. Due to the extremely small positive dataset size in triplex lncRNA prediction, *i.e*. 531, we also applied oversampling on this dataset. Because the positive data in triplex lncRNA prediction are collected from two sources whose amounts are in a ratio around 2.5 to 1 (triplexlncRNA to reported triplex lncRNA), to force the model to learn the global features for all positive data rather than the common features for the data from the majority type, *i.e.* triplexlncRNA, we assigned more weights to the weak type data, *i.e.* reported triplex lncRNA, during augmenting the positive data in the training process, and we named this practice as the weighted bagging strategy.

### Model evaluation

To demonstrate the model performances, the evaluation criteria of accuracy (Acc), sensitivity (Sn), specificity (Sp), and AUROC are used. Besides, criteria in evaluating imbalance data are also adopted, including AUPRC, F1-score, and harmonic mean (Hm). The equations for calculating the above criteria are listed below.

3 $$Accuracy\, \left( {Acc} \right) = \frac{TP + TN}{{TP + TN + FP + FN}}$$

4 $$Sensitivity \,\left( {Sn} \right) = \frac{TP}{{TP + FN}}$$

5 $$Specificity \,\left( {Sp} \right) = \frac{TN}{{TN + FP}}$$

6 $$Harmonic\, mean\, of\,Sn\, and\,Sp \,\left( {Hm} \right) = \frac{2 \times Sn \times Sp}{{Sn + Sp}}$$

7 $$F1{\text{-}}score = \frac{2 \times PRE \times Sn}{{PRE + Sn}}, \quad where\,PRE = \frac{TP}{{TP + FP}}$$

where FN, FP, TN, and TP denote the number of false negative, false positive, true negative, and true positive, respectively.

## Results

### Initial training of TriplexFPP

Each input sequence is represented into a fix-length 90-dim vector by considering both k-mer ($k = 1, 2, 3,\, number\, of\, feature = 84$) and kmerscore ($k = 1 - 6,\, number\, of\, feature = 6$) features. The influences of the k-mer and kmerscore features to TriplexFPP can be found at Additional file 1: Fig. S1. We use the mean k-mer feature values from training data to calculate the kmerscore features of both training and test data in the corresponding split to exclude any information from test data.

**Fig. 1:**
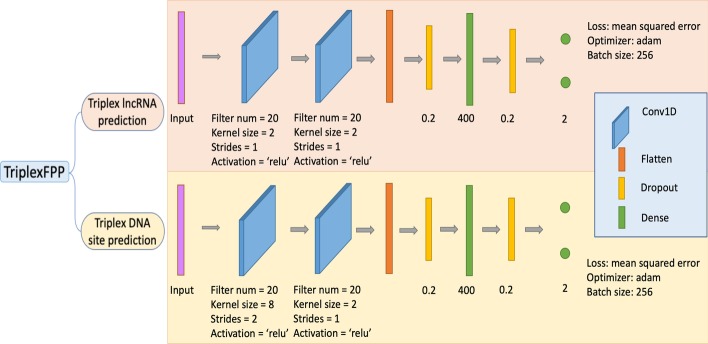
The architecture of TriplexFPP. TriplexFPP is composed of two models, the corresponding model architecture and parameters are shown

The parameters in TriplexFPP, such as the number of convolutional layer, the kernel size, the activation function, etc. are determined according to the corresponding random split datasets in two individual models. Each time we change the value of one parameter while keeping other parameters fixed, and then select the one that achieves the highest value of Sn as the final choice for that parameter. The setting of the parameters and the corresponding performances for triplex lncRNA prediction model and triplex DNA sites prediction model are shown in Additional file 1: Figs S2 and S3 separately. The detailed architectures and parameters for TriplexFPP can be found in Fig. 1.

### Evaluation of triplex lncRNA prediction

In this section, we evaluate the triplex lncRNA prediction model in TriplexFPP regard to its ability in predicting the most likely triplex-forming lncRNA from the lncRNAs owning triplex forming capability predicted by the computation methods.

**Fig. 2:**
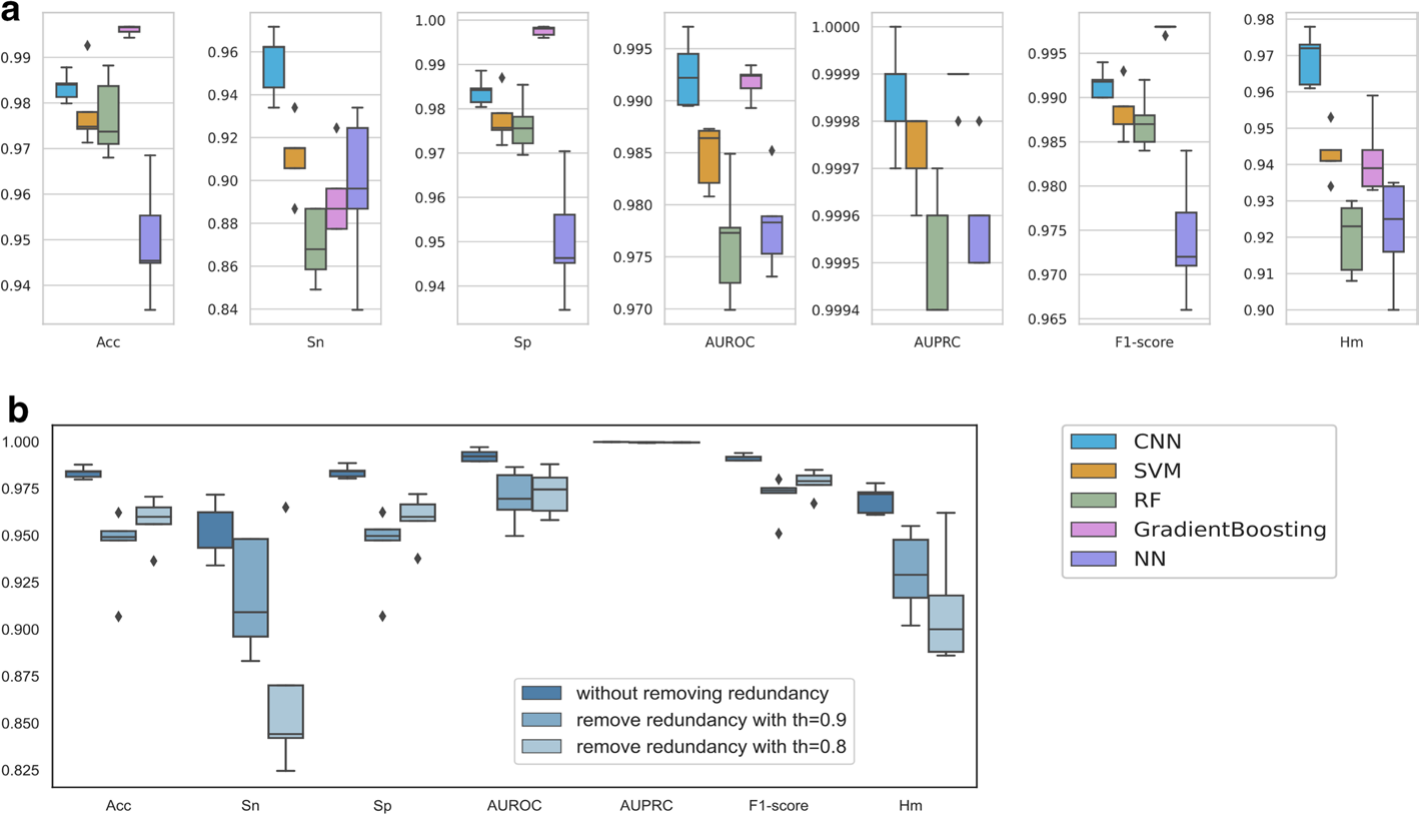
Evaluation of lncRNA triplex prediction model in TriplexFPP. **a** The box and whisker plot of the fivefold cross validation for CNN and 4 baseline modes (SVM, RF, Gradient Boosting, and NN). **b** The comparisons of the fivefold cross validation performances among data without removing redundancy, removing redundancy with threshold 0.9, and removing redundancy with threshold 0.8

We first visualize the nucleotide compositions of lncRNA sequences in our positive dataset (triplexlncRNA and reported triplex lncRNA) and negative dataset (Additional file 1: Fig. S4). The nucleotide compositions for the reported triplex lncRNAs and the negative lncRNAs are more consistent, from which nucleotide A and T taking up heavier percentages; whereas the triplexlncRNA follows a different pattern, whose sequences are mainly CG rich. Because the amount of triplexlncRNA is larger than that of reported triplex lncRNA in our positive dataset, to ensure the model to learn the high-level features for all positive data rather than the sequence composition features of triplexlncRNA, we assign more weights to the reported triplex lncRNA when augmenting the positive data in the training process. We triple the positive training data with the weighted bagging strategy, where two-thirds of them is bagged from the original positive training data directly and one-third of them is extra bagged from the reported triplex lncRNA. We compare our method with baseline models like Deep Neural Network (NN), Support Vector Machine (SVM), Random Forest (RF), and Gradient Boosting. The parameters for the NN model are determined as the ones with the best Sn value among several choices on random split training and test datasets, and the parameters for other baseline models are determined as the optimal ones by cross-validated grid-search over a parameter grid based on the criteria of Recall. The candidate parameters and the final determined parameters for each baseline model are recorded in Additional file 1: Table S1. The box and whisker plots for fivefold cross validation are demonstrated for all models in Fig. 2a, where the negative training data are randomly selected as the same amount of augmented positive training data from the 4 training folds in each validation.

With the mission of finding the most likely triplex-forming lncRNAs in practice, although Gradient Boosting method realizes the best Acc and F1-score values, its Sn values mainly concentrate between 87.74 and 92.45%. Conversely, the Sn values for CNN model range from 93.40 to 97.17%, concentrating at a high-value region, which indicates its superiority to baseline models on the model performance. Besides, the overall high values of other evaluation metrices in CNN model, *e.g.* 98.35% of average Acc, 0.9926 of average AUROC, 0.9999 of average AUPRC, 0.992 of average F1-score, and 0.969 of average Hm, further illustrate the effectiveness of our CNN model in TriplexFPP in the lncRNA triplex forming potential prediction.

However, one issue for the above fivefold cross validation is that the high performance may be caused by the high data similarity between training and test. In our positive dataset, the 531 samples relate to 135 genes (Additional file 1: Figure S5), where 57 genes owning two or more variants. The different variants from the same lncRNA gene can share high sequence composition similarities, thereby leading to a good prediction result. To evaluate whether the model is powerful enough in predicting lncRNA triplex forming potential, we further execute fivefold cross validation on the datasets with removed redundancy and carry out the leave-out validation.

We compare the performances of CNN model on datasets without removing redundancy, removing redundancy with threshold of 0.9, and removing redundancy with threshold of 0.8. The results are demonstrated in Fig. 2b. Although the average performances on the data with removed redundancy are slightly lower than that of without removing redundancy, the values in evaluation matrices on the removed redundancy datasets remain at high levels. For example, in removed redundancy datasets with threshold of 0.9, the AUROC and AUPRC values range from 0.9637 to 0.9880 and 0.9993 to 0.9998 separately; in removed redundancy datasets with threshold of 0.8, the AUROC and AUPRC values range from 0.9497 to 0.9809 and 0. 9994 to 0.9998 separately.

For the leave-out validation, we select four lncRNAs with the most amounts of variants as the test data, including MIR100HG, PVT1, LINC00963, and MEG3. Their variants amounts are 87, 73, 52, and 46, respectively. The four lncRNAs follow different data sources, PVT1 and LINC00963 belong to triplexlncRNA, and MIR100HG and MEG3 belong to reported triplex lncRNA. In each leave-out validation, we select one lncRNA and use all of its variants as positive test data, while using all the remaining positive lncRNAs as the training data. The training and test process are repeated 5 times, each time the negative training data are randomly selected as the same amount with positive training data from one of the fivefold cross validation split above, and the negative test data are the above fivefold cross validation test data.

When leaving lncRNA PVT1 and LINC00963 out, our model predicts all their variants correctly as positive. The average AUROC values for PVT1 and LINC00963 are 0.9996 and 0.9968 separately. However, when leaving lncRNA MIR100HG and MEG3 out, their average AUROC values are 0.6594 and 0.3220 separately, which are a bit low. One possible reason for the different performances between triplexlncRNA and reported triplex lncRNAs is that, in triplexlncRNA, we adopt the variants which are overlapped with experimentally verified triplex forming regions; whereas in the reported triplex lncRNA, we adopt all the variants of that gene, however, maybe not all of these variants could form triplex in practice. Interestingly, when we only use six kmerscore features to train leave-out model for MEG3, its average AUROC value could increase to 0.7610, but this phenomenon is not found when leaving MIR100HG out.

### Evaluation of triplex DNA sites potential prediction

**Fig. 3:**
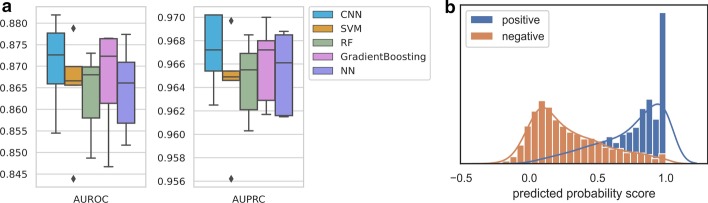
Evaluation of triplex DNA sites potential prediction model in TriplexFPP. **a** The box and whisker plot of fivefold cross validation for CNN and baseline models. **b** The visualization of the distribution for predicted probability score of the first fold validation data

We use the fivefold cross validation to evaluate the performance of triplex DNA sites potential prediction model in TriplexFPP, and compare it with other baseline models (Fig. 3a). The determination of the parameters for baseline models in predicting triplex DNA sites follows the same procedure as that of triplex lncRNA prediction. The candidate parameters and the final determined parameters for each baseline model are recorded in Additional file 1: Table S2. The overall performance of CNN is better than that of baseline models, whose average AUROC and AUPRC values are 0.8705 and 0.9671 separately; whereas for baselines methods, the average values of AUROC located in the scope of 0.8635 to 0.8667, and the average values of AUPRC are from 0.9642 to 0.9660, respectively. We then visualize the predicted probability scores of CNN model in each fold (Fig. 3b and Additional file 1: Fig. S6). Although some samples are wrongly predicted, the predicted probability scores for most positive data concentrate at around value of 1. This phenomenon indicates that our model can predict most data correctly with high confidence.

Besides, the region of 649–708 in HOTAIR sequence is verified to form DNA:RNA triplex [10], our model correctly predicts this site as the triplex forming type. Overall, with the limited data, our results demonstrate that TriplexFPP can effectively distinguish the in vivo assay defined triplex-forming DNA sites from those background sites with only nucleotide sequence features as the input.

### TriplexFPP model interpretation

**Fig. 4:**
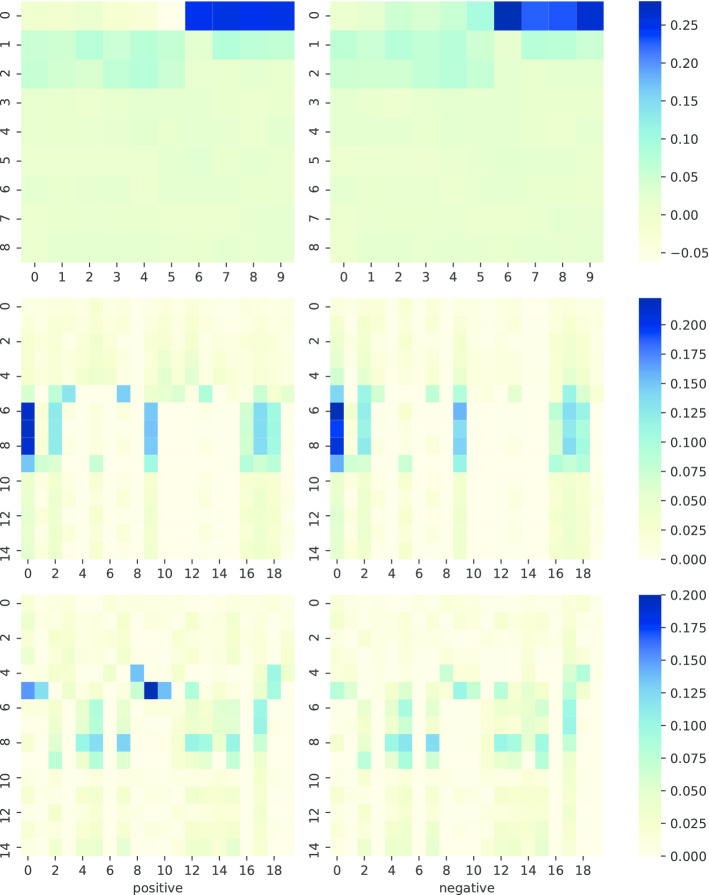
The average feature values in each class of triplex lncRNA prediction model. Top: original features (the 90-dim features are reshaped to 9*10), middle: features after trained with one CNN layer (x-axis: filter, y-axis: convolution values the 1st to the 15th), and bottom: features after trained with two CNN layers (x-axis: filter, y-axis: convolution values the 1st to the 15th); left: positive data, right: negative data

Take the first fold validation in two models in TriplexFPP as examples, we plot the average feature values for each class in the format of heatmap before training (original features), trained after one CNN layer, and trained after two CNN layers in Fig. 4 and Additional file 1: Fig. S7. The kmerscore features (the first six features in original features) show obvious differences in two classes, which indicate that the nucleotide compositions have different preferences with regard to the positions in positive and negative data. The kmerscore features also lead to different convolution values after trained with only one CNN layer. However, for the remaining features, the differences of their convolutional values between two classes do not show noticeable differences until trained after 2 CNN layers.

### In cis/in trans triplex-forming lncRNA: an exploration

The lncRNA can form triplex structures with DNA both *in cis* and *in trans*, but if differences exist between *cis* and *trans* targeting of triplexes lncRNAs remains unknown [28]. In this work, we explore the *cis* and *trans* targeting of triplexes lncRNAs following two data sources.

From the TDF results between each triplexlncRNA and TriplexDNA, 238 lncRNAs in triplexlncRNAs show both *in cis* and *in trans* interactions with DNA, whose *in cis* binding numbers range from 1 to 2450; whereas the other 141 lncRNAs only show *in trans* interactions with DNA (Additional file 1: Fig. S8). Moreover, for a certain lncRNA gene, its variants may show different binding patterns. Among all 130 genes related to triplexlncRNAs, 26 genes contain variants either belong to the type of *in cis* and *in trans* interactions or only *in trans* interactions (Additional file 1: Figs. S9 and S10).

Besides, for those reported triplex lncRNA, we collect their binding information from the published work in Additional file 1: Table S3 [7, 8, 10, 11, 23, 29]. According to the corresponding experiments, the lncRNA HOTAIR, MEG3, and MIR100HG show *in trans* binding, and PARTICLE and FENDRR show *in cis* binding.

## Discussion

LncRNA can exert functions via interacting with DNA. Among all kinds of interactions, DNA:RNA triplex formation is still less understood to us due to the limited number of validation assays. Although varieties of canonical rule-based computational methods have been developed to predict the triplex forming potential for lncRNA and DNA sites, they identify a large number of lncRNAs which can form triplex. However, the limited number of experimentally verified data indicates that maybe not all of them can form triplex in practice. Besides, those computational methods only theoretically calculate the triplex potential, while do not consider any in vivo and in vitro verified data.

Trained with the data obtained from in vitro and in vivo assays, our newly developed program, namely TriplexFPP, exhibits good prediction performances. Its triplex lncRNA prediction model works effectively by achieving high average scores of evaluation matrices in the fivefold cross validation. For example, in the removed redundancy datasets with threshold 0.8, the average cross fold validation value of Acc, AUROC, AUPRC, f1-score, and Hm are 95.28%, 0.9649, 0.9996, 0.976, and 0.904, respectively. Besides, the triplex DNA sites potential prediction model in TriplexFPP also works effectively. In the fivefold cross validation, its average AUROC and AUPRC values are 0.8705 and 0.9671 separately. And most data are predicted correctly with high confidences.

We also summarized the *cis* and *trans* targeting of triplexes lncRNAs following the different data sources collected in this work, which may provide some insights to the exploration of lncRNA *cis* and *trans* binding mechanisms.

However, one limitation for this work is that the positive data amount is small. And also, some data in our negative class may belong to the positive but are not yet verified. Therefore, we expect more data to be explored to help implementing this tool. Besides, a small fraction lncRNA in our collected data may belong to the R-Loop forming type, which may influence the results somehow.

## Conclusion

We proposed a deep learning based program in DNA:RNA triplex formation prediction, namely TriplexFPP. TriplexFPP predicts the most likely triplex-forming lncRNAs from all the lncRNAs with computationally defined triplex forming capacities, and it also predicts the potential of a DNA site to become a triplex. TriplexFPP narrows the scope of possible lncRNAs in forming triplex compared to those mathematic statistic methods. We expect the TriplexFPP can provide insights and references to help to decipher the codes of the lncRNA functions.

### Availability and requirements

- *Project name*: TriplexFPP.
- *Project home page*: https://github.com/yuuuuzhang/TriplexFPP
- *Operating system(s)*: Platform independent
- *Programming language*: Python
- *Other requirements*: python3, tensorflow 1 (> = 1.12)
- *License*: GNU General Public License v3.0
- *Any restrictions to use by non-academics*: Not applicable.

## Supplementary information

**Additional file 1.** Supplementary materials (Supplementary Tables S1–S3, Supplementary Figures S1–S10).

## Data Availability

The datasets used in this work can be found at https://github.com/yuuuuzhang/TriplexFPP_data, and the code can be found at https://github.com/yuuuuzhang/TriplexFPP.

## Abbreviations

TriplexFPP: Triplex forming potential prediction
AUROC: Area under the ROC
AUPRC: Area under the PRC
Acc: Accuracy
Sn: Sensitivity
Sp: Specificity
Hm: Harmonic mean
DBD: DNA binding domain
TFO: Triplex forming oligonucleotide
DBS: DNA binding site
NN: Deep neural network
SVM: Support vector machine
RF: Random forest
