# Supplementary material for: Deep learning based DNA:RNA triplex forming potential prediction
Source: BMC Bioinformatics. 2020 Nov 12;21:522. doi: 10.1186/s12859-020-03864-0 (PMC7663897; doi:10.1186/s12859-020-03864-0)
Supplement: Supplementary file 1 — Additional file 1. Supplementary materials (Supplementary Tables S1–S3, Supplementary Figures S1–S10). [file 12859_2020_3864_MOESM1_ESM.docx]

**Supplementary Materials for** Deep learning based DNA:RNA triplex forming potential prediction

Yu Zhang^1^, Yahui Long^2^ and Chee Keong Kwoh^1,*^

^1^School of Computer Science and Engineering, Nanyang Technological University, 639798, Singapore, ^2^College of Computer Science and Electronic Engineering, Hunan University, Changsha 410000, China.

* To whom correspondence should be addressed. Tel: +65 6790 6057; Fax: +65 6792 6559; Email: asckkwoh@ntu.edu.sg

Tables

[Table S1. The candidate and determined parameters for baseline models in predicting triplex lncRNA. 2](#_Toc50901908)

[Table S2. The candidate and determined parameters for baseline models in predicting triplex DNA sites. 3](#_Toc50901909)

[Table S3. The binding information for reported triplex lncRNA. 4](#_Toc50901910)

Figures

[Figure S1. Determining the features in TriplexFPP. 5](#_Toc50901911)

[Figure S2. TriplexFPP triplex lncRNA prediction model network parameter determination. 6](#_Toc50901912)

[Figure S3. TriplexFPP triplex DNA site prediction model network parameter determination. 7](#_Toc50901913)

[Figure S4. The nucleotide frequency distribution in positive data (both triplexlncRNA and reported triplex lncRNA) and negative data in lncRNA triplex prediction model. 8](#_Toc50901914)

[Figure S5. The demonstration of the number of variants for each gene. 8](#_Toc50901915)

[Figure S6. The distribution for predicted probability scores of the cross-fold validation test data. 9](#_Toc50901916)

[Figure S7. The average values in each class. 10](#_Toc50901917)

[Figure S8. Statistic of in cis & in trans lncRNAs in TriplexlncRNA. 11](#_Toc50901918)

[Figure S9. Statistic of in cis & in trans lncRNAs in TriplexlncRNA. 11](#_Toc50901919)

[Figure S10. Gene names and number of variants in two types of data in in cis / in trans lncRNA prediction. 11](#_Toc50901920)

# Table S1. The candidate and determined parameters for baseline models in predicting triplex lncRNA. The parameters for SVM, RF, and GB models are searched by sklearn.model_selection.GridSearchCV function in python with 5 folds and ‘recall’ scoring method.

| Model | Candidate parameters | Determined parameters |
| --- | --- | --- |
| SVM | C = { 2, 5, 10, 15, 20, 25, 30}  gamma = { 0.1 ,0.2, 0.5, 1, 10,'scale'} | kernel=’rbf’  C=25  gamma=’scale’ |
| RF | min_samples_leaf = {3, 5, 7, 9}  max_depth = {4, 5, 6, 7}  min_samples_split = {2, 4, 6, 8, 10, 20, 40, 60}  n_estimators = {100, 200, 300, 400, 500} | min_samples_leaf=3  max_depth=7  min_samples_split=2  n_estimators=200  max_features='sqrt' |
| GB | min_samples_leaf = {3, 5, 7, 9}  max_depth = {4, 5, 6, 7}  min_samples_split = {2, 4, 6, 8, 10, 20, 40, 60}  n_estimators = {100, 200, 300, 400, 500}  subsample = {0.7, 0.8, 0.9, 1}  learning_rate = {0.1, 0.05, 0.01, 0.005, 0.001} | min_samples_leaf=9  max_depth=5  min_samples_split=4  n_estimators=200  subsample=1  learning_rate=0.1  max_features='sqrt' |
| NN | Number_of_layer = {1 , 2, 3}  Dense_size = {16, 32, 64, 256}  Activation_function = {relu, tanh, sigmoid, softmax}  Dropout_rate = {0.2, 0.3, 0.4, 0.5}  Optimizer = {adam, sgd, adadelta, adagrad, adamax} | Number_of_layer = 2  Layer1_dense_size = 64  Layer2_dense_size = 16  Activation_function = relu  Dropout_rate = 0.2  Optimizer = adam |

# Table S2. The candidate and determined parameters for baseline models in predicting triplex DNA sites. The parameters for SVM, RF, and GB models are searched by sklearn.model_selection.GridSearchCV function in python with 5 folds and ‘recall’ scoring method.

| Model | Candidate parameters | Determined parameters |
| --- | --- | --- |
| SVM | C = { 2, 5, 10, 15, 20, 25, 30}  gamma = { 0.1 ,0.2, 0.5, 1, 10,'scale'} | kernel=’rbf’  C=2  gamma=1 |
| RF | min_samples_leaf = {3, 5, 7, 9}  max_depth = {4, 5, 6, 7}  min_samples_split = {2, 4, 6, 8, 10, 20, 40, 60}  n_estimators = {100, 200, 300, 400, 500} | min_samples_leaf=7  max_depth=7  min_samples_split=2  n_estimators=500  max_features='sqrt' |
| GB | min_samples_leaf = {3, 5, 7, 9}  max_depth = {4, 5, 6, 7}  min_samples_split = {2, 4, 6, 8, 10, 20, 40, 60}  n_estimators = {100, 200, 300, 400, 500}  subsample = {0.7, 0.8, 0.9, 1}  learning_rate = {0.1, 0.05, 0.01, 0.005, 0.001} | min_samples_leaf=9  max_depth=4  min_samples_split=60  n_estimators=100  subsample=0.9  learning_rate=0.05  max_features='sqrt' |
| NN | Number_of_layer = {1 , 2, 3}  Dense_size = {16, 32, 64, 256}  Activation_function = {relu, tanh, sigmoid, softmax}  Dropout_rate = {0.2, 0.3, 0.4, 0.5}  Optimizer = {adam, sgd, adadelta, adagrad, adamax} | Number_of_layer = 2  Layer1_dense_size = 64  Layer2_dense_size = 16  Activation_function = relu  Dropout_rate = 0.2  Optimizer = adam |

# Table S3. The binding information for reported triplex lncRNA.

| lncRNA | lncRNA chromosome | Bound DNA name and chromosome | type |
| --- | --- | --- | --- |
| HOTAIR | chr12 | PCDH7 chr4  HOXB2 chr17 [1,6] | *In trans* |
| MEG3 | chr14 | TGF-β chr3 [2,6] | *In trans* |
| PARTICL | chr2 | MAT2A chr2 [3,6] | *In cis* |
| MIR100HG | chr11 | P27(CDKN1B) chr12 [4] | *In trans* |
| FENDRR | chr16 | FOXF1 chr16 [5] | *In cis* |

# Figure S1. Determining the features in TriplexFPP. Left: triplex lncRNA prediction model, right:: triplex DNA sites prediction model.


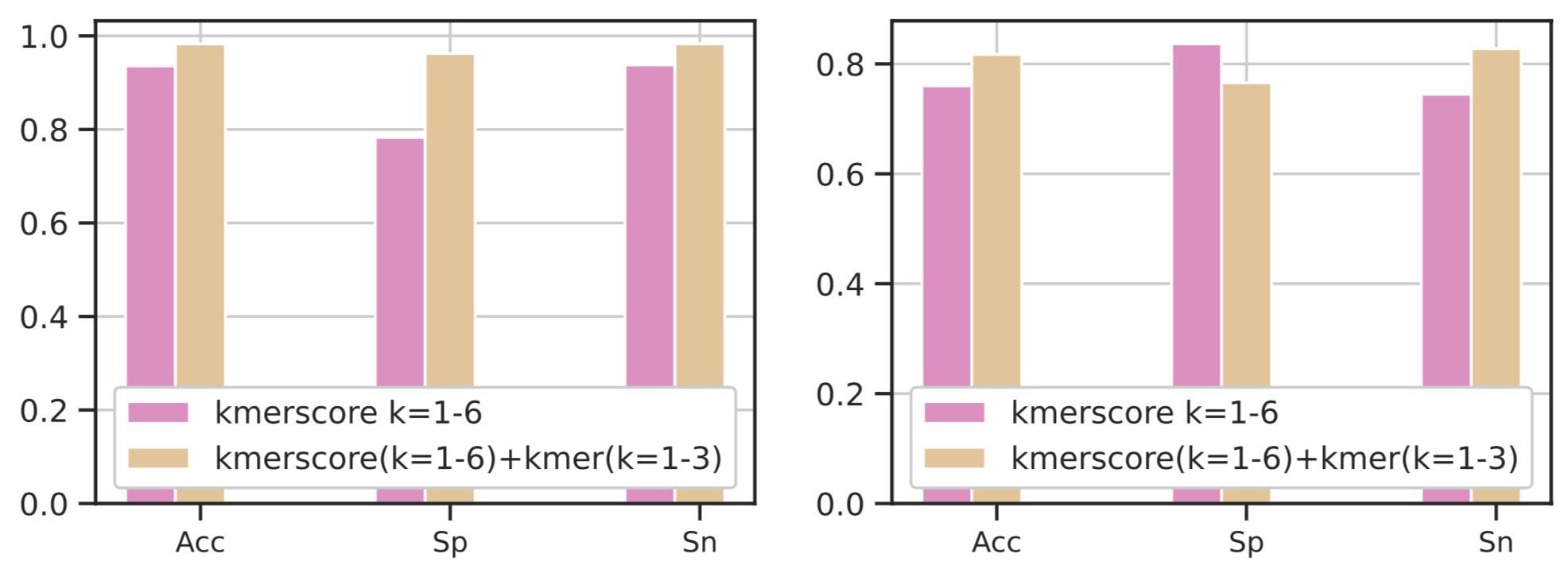


# Figure S2. TriplexFPP triplex lncRNA prediction model network parameter determination. (a) Accuracies achieved with different numbers of convolution layers. (b) Accuracies achieved with different choices of activation functions. (c) Accuracies achieved with different choices of kernel and stride size in the 1^st^ convolution layer. (d) Accuracies achieved with different choices of kernel and stride size in the 2^nd^ convolution layer. (e) Accuracies achieved with different choices of filter number in the 1^st^ and 2^nd^ convolution layer. (f) Accuracies achieved with different choices of dropout rates. (g) Accuracies achieved with different choices of optimizers. (h) The training and test accuracies achieved with different choices of training epochs, the best test accuracy is marked by dash line and the corresponding number of training epoch (110) is adopted in constructing model.


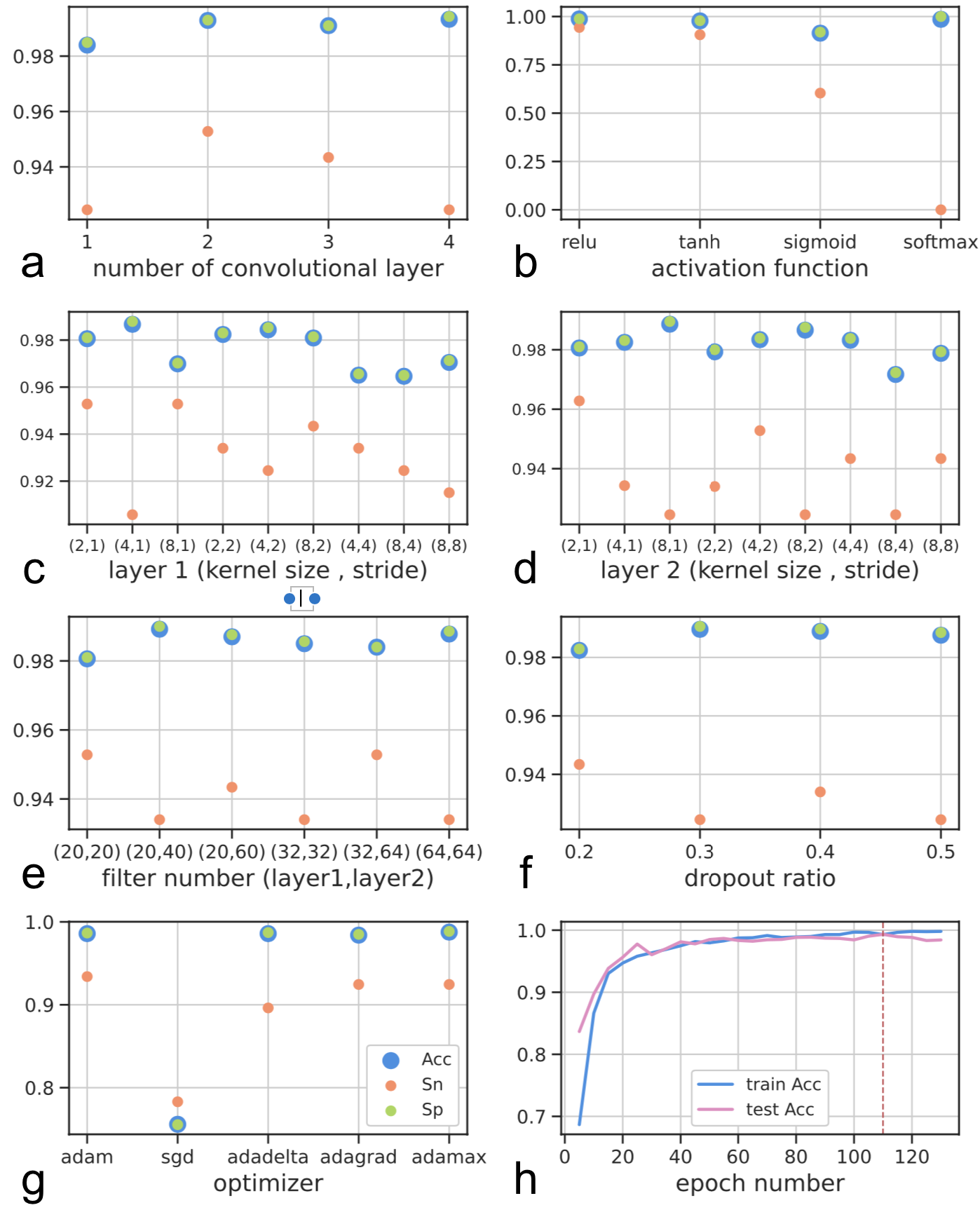


# Figure S3. TriplexFPP triplex DNA site prediction model network parameter determination. (a) Accuracies achieved with different numbers of convolution layers. (b) Accuracies achieved with different choices of activation functions. (c) Accuracies achieved with different choices of kernel and stride size in the 1^st^ convolution layer. (d) Accuracies achieved with different choices of kernel and stride size in the 2^nd^ convolution layer. (e) Accuracies achieved with different choices of filter number in the 1^st^ and 2^nd^ convolution layer. (f) Accuracies achieved with different choices of dropout rates. (g) Accuracies achieved with different choices of optimizers. (h) The training and test accuracies achieved with different choices of training epochs, the best test accuracy is marked by dash line and the corresponding number of training epoch (75) is adopted in constructing model.


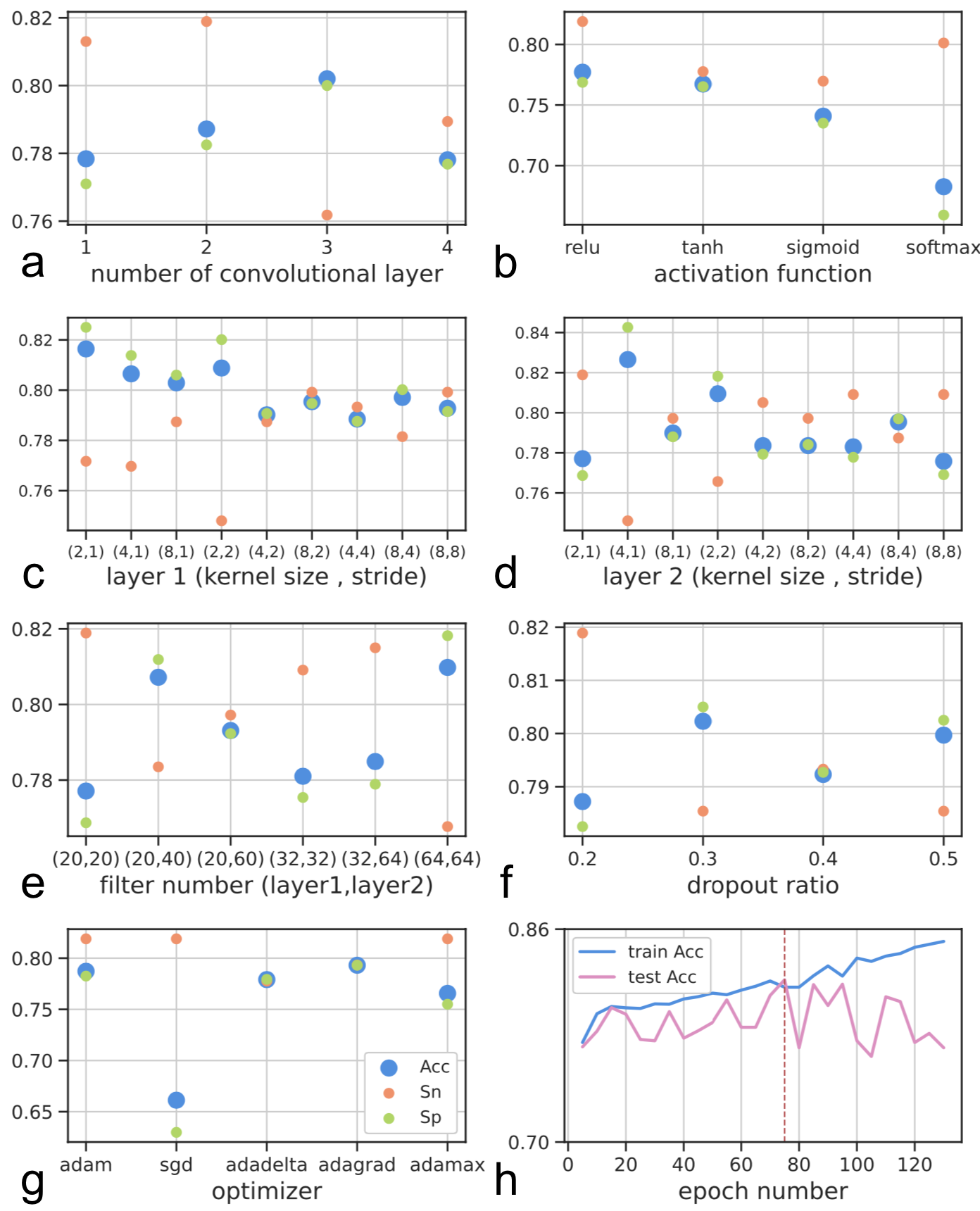


# Figure S4. The nucleotide frequency distribution in positive data (both triplexlncRNA and reported triplex lncRNA) and negative data in lncRNA triplex prediction model.


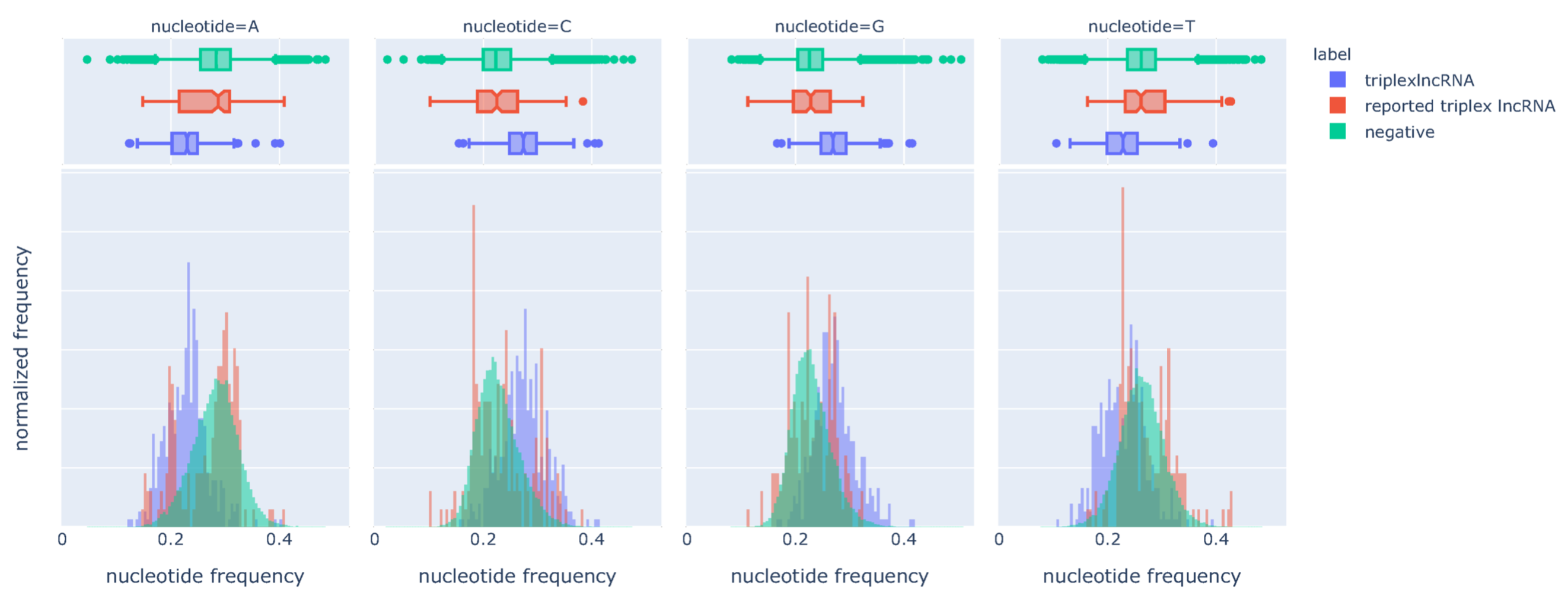


# Figure S5. The demonstration of the number of variants for each gene, the light color to dark color means a small variant number to a large variant number, outer: triplexlncRNA, inner: reported triplex lncRNA.


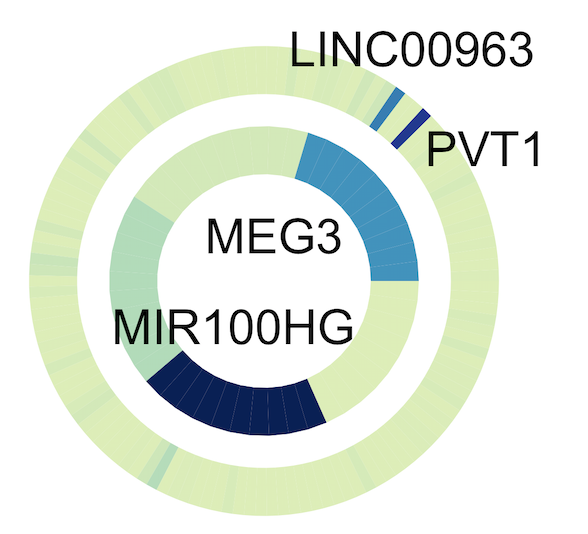


# Figure S6. The distribution for predicted probability scores of the cross-fold validation test data. From upper left to lower right: fold 2 to fold 5.


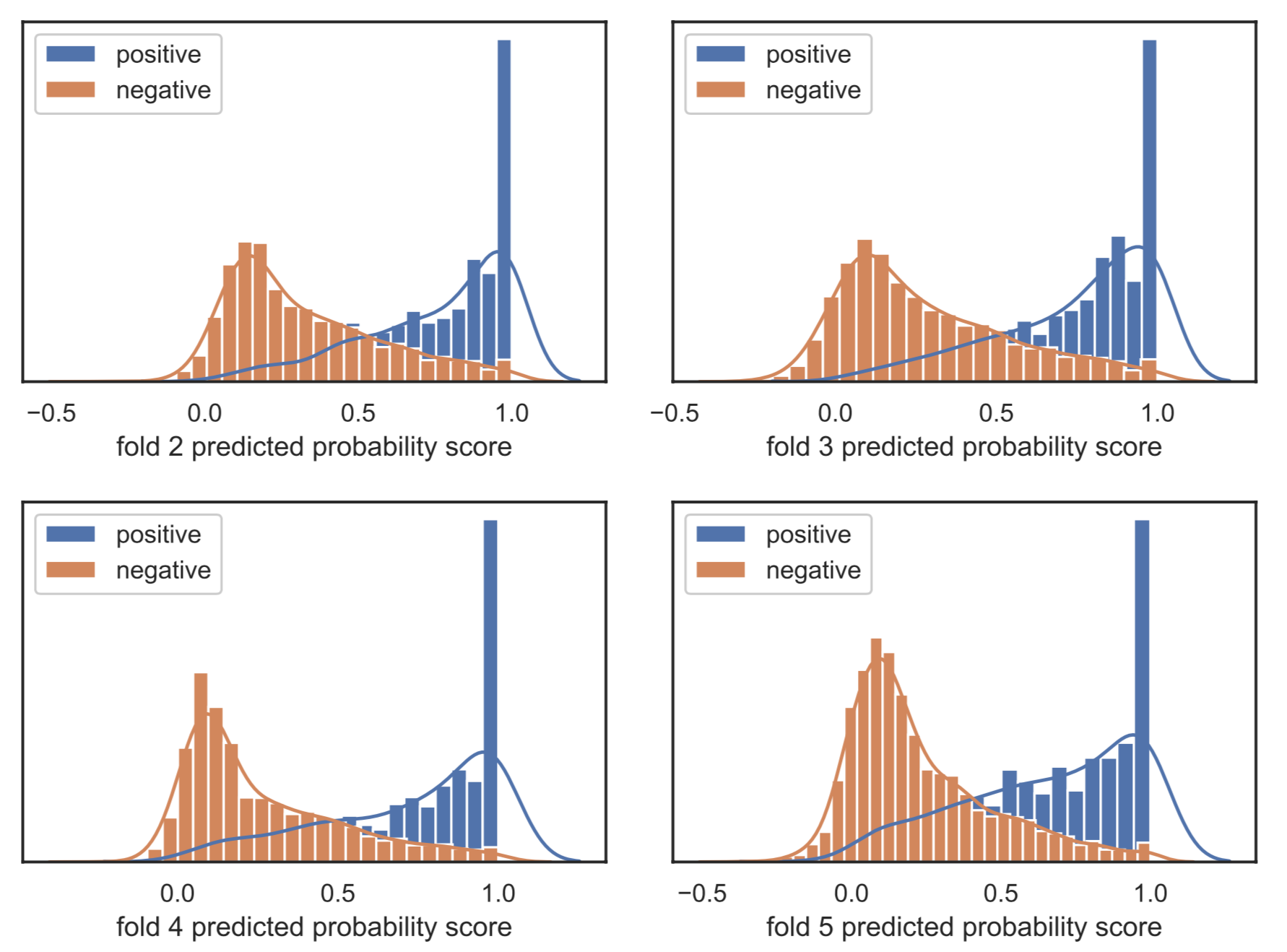


# Figure S7. The average feature values in each class of triplex DNA sites prediction model. Top: original features (the 90-dim features are reshaped to 9*10), middle: features after trained with one CNN layer (x-axis: filter, y-axis: convolution values the 1st to the 15th), and bottom: features after trained with two CNN layers (x-axis: filter, y-axis: convolution values the 1st to the 15th); left: positive data, right: negative data.


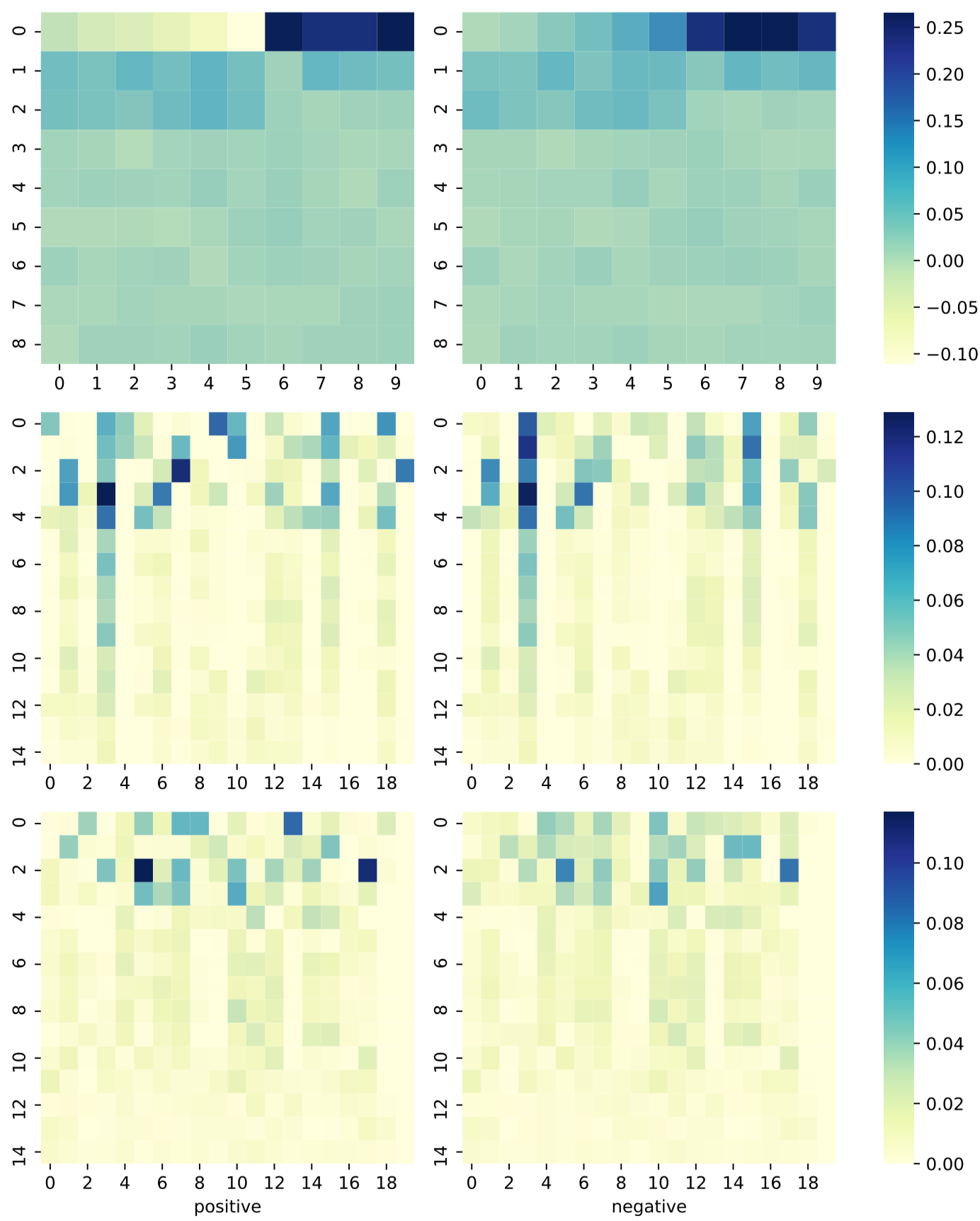


# Figure S8. Statistic of in cis & in trans lncRNAs in TriplexlncRNA. Left: the number of lncRNAs in 2 classes, right: the distribution of the number of in cis binding sites for in cis & in trans lncRNAs.


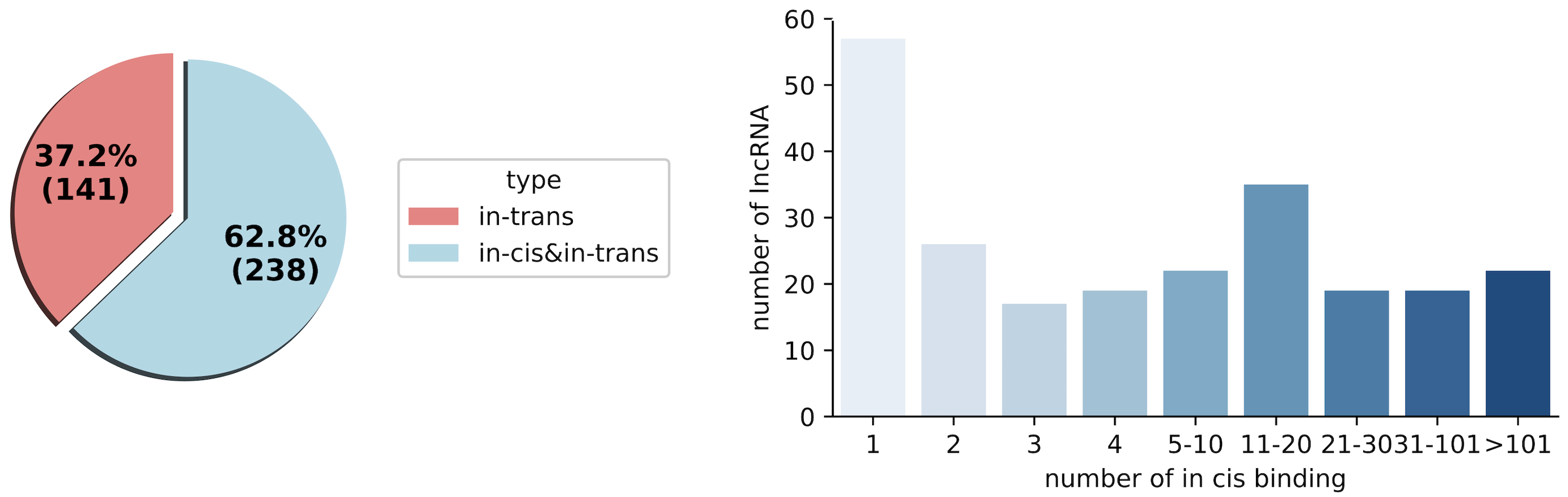


# Figure S9. Statistic of *in cis & in trans* lncRNAs in TriplexlncRNA. Left: gene numbers in each type, right: variant numbers of PVT1 and LINC00963 in two classes.


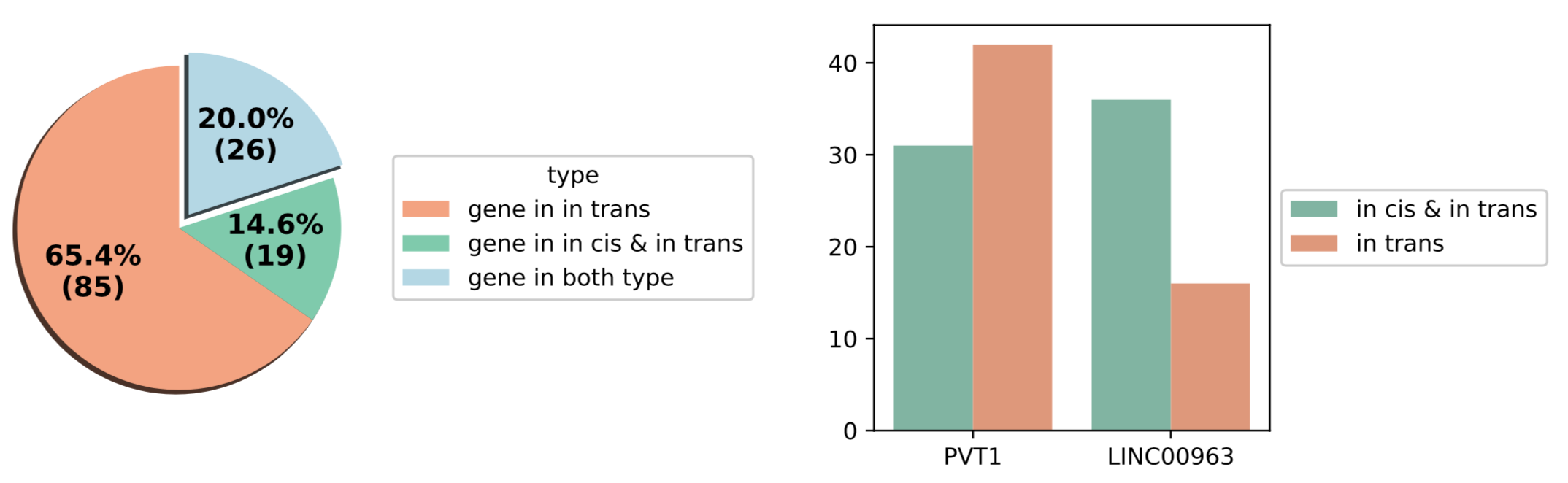


# Figure S10. Gene names and number of variants in two types of data in in cis / in trans lncRNA prediction.

1. Kalwa M, Hänzelmann S, Otto S, Kuo CC, Franzen J, Joussen S, Fernandez-Rebollo E, Rath B, Koch C, Hofmann A, Lee SH. The lncRNA HOTAIR impacts on mesenchymal stem cells via triple helix formation. Nucleic acids research. 2016 Dec 15;44(22):10631-43.
2. [2] Mondal T, Subhash S, Vaid R, Enroth S, Uday S, Reinius B, Mitra S, Mohammed A, James AR, Hoberg E, Moustakas A. MEG3 long noncoding RNA regulates the TGF-β pathway genes through formation of RNA–DNA triplex structures. Nature communications. 2015 Jul 24;6:7743.
3. O’Leary VB, Ovsepian SV, Carrascosa LG, Buske FA, Radulovic V, Niyazi M, Moertl S, Trau M, Atkinson MJ, Anastasov N. PARTICLE, a triplex-forming long ncRNA, regulates locus-specific methylation in response to low-dose irradiation. Cell reports. 2015 Apr 21;11(3):474-85.
4. Wang S, Ke H, Zhang H, Ma Y, Ao L, Zou L, Yang Q, Zhu H, Nie J, Wu C, Jiao B. LncRNA MIR100HG promotes cell proliferation in triple-negative breast cancer through triplex formation with p27 loci. Cell death & disease. 2018 Jul 24;9(8):1-1.
5. Navarro C, Cano C, Cuadros M, Herrera-Merchan A, Molina M, Blanco A. A mechanistic study of lncRNA Fendrr regulation of FoxF1 lung cancer tumor supressor. InInternational Conference on Bioinformatics and Biomedical Engineering 2016 Apr 20 (pp. 781-789). Springer, Cham.
6. Li Y, Syed J, Sugiyama H. RNA-DNA triplex formation by long noncoding RNAs. Cell chemical biology. 2016 Nov 17;23(11):1325-33.
